# Association between asymmetric dimethylarginine and sarcopenia in community-dwelling older women

**DOI:** 10.1038/s41598-023-32046-0

**Published:** 2023-04-04

**Authors:** Miyuki Yokoro, Naoto Otaki, Megumu Yano, Tomomi Imamura, Norikazu Tanino, Keisuke Fukuo

**Affiliations:** 1 Department of Dietary Life and Food Sciences, Junior College Division, Mukogawa Women’s University, 6-46 Ikebiraki-Cho, Nishinomiya, Hyogo 663-8558 Japan; 2 Research Institute for Nutrition Sciences, Mukogawa Women’s University, 6-46 Ikebiraki-Cho, Nishinomiya, Hyogo 663-8558 Japan; 3 Department of Food Sciences and Nutrition, School of Food Sciences and Nutrition, Mukogawa Women’s University, 6-46 Ikebiraki-Cho, Nishinomiya, Hyogo 663-8558 Japan; 4 Department of Innovative Food Sciences, School of Food Sciences and Nutrition, Mukogawa Women’s University, 6-46 Ikebiraki-Cho, Nishinomiya, Hyogo 663-8558 Japan

**Keywords:** Risk factors, Geriatrics, Epidemiology, Preclinical research

## Abstract

Asymmetric dimethylarginine (ADMA) is an endogenous inhibitor of endothelium nitric oxide synthesis and causes endothelial dysfunction that may be related to sarcopenia. However, the association between ADMA and sarcopenia has not been studied. We evaluated the correlations between plasma ADMA levels and sarcopenia in community-dwelling older women. In total, 144 community-dwelling older women participated in this study. Plasma ADMA levels were measured using a competitive enzyme-linked immunosorbent assay. Skeletal muscle mass, measured in terms of bioimpedance and grip strength, was used to assess sarcopenia. Plasma ADMA levels were significantly higher in participants with sarcopenia than in those without sarcopenia. Through receiver-operating characteristic curve analysis, the cutoff value of plasma ADMA level for sarcopenia was estimated at 0.57 μM. Sarcopenia was significantly more prevalent in participants with higher plasma ADMA levels than in those with lower plasma ADMA levels. According to logistic regression analysis, the crude odds ratio of higher plasma ADMA levels in participants with sarcopenia was 4.57 (95% confidence interval, 1.82–11.47; *p* = 0.001). Reductions in the skeletal muscle mass index over 2 years were significantly greater in participants with higher plasma ADMA levels. In conclusion, plasma ADMA levels were significantly associated with sarcopenia in community-dwelling older women.

## Introduction

Sarcopenia is characterized by an age-related loss of muscle mass, muscle strength, and/or physical performance^1^. It contributes to the development of frailty and is associated with poor clinical outcomes, mortality, functional disability, falls, hip fractures, and hospitalization^2–4^. Factors found to increase the risk of sarcopenia include aging, physical inactivity, malnutrition, poor dental health, and chronic diseases such as cardiovascular diseases, diabetes, hypertension, and dementia^2,5–8^. In particular, multiple physiological changes associated with aging have been found to contribute to the development of sarcopenia. These include protein synthesis abnormalities and degradation, cellular senescence, oxidative stress, mitochondrial dysfunction, low-grade inflammation, inadequate nutrition, hormonal changes, and satellite cell dysfunction. However, the pathophysiological mechanisms responsible for sarcopenia have not yet been fully elucidated^9^. Recent studies have found a relationship between endothelial dysfunction and age-related loss of muscle mass and grip strength^10,11^.

Asymmetric dimethylarginine (ADMA) is an endogenous amino acid that competitively inhibits the endothelial synthesis of nitric oxide (NO), which is a putative vasodilator^12^. Increased ADMA levels cause endothelial dysfunction and are a known risk factor for cardiovascular diseases^13^. Relationships have been reported between blood ADMA levels and physical performance, muscle strength, and gait speed in dialysis patients and older individuals, suggesting that ADMA plays a role in the development of sarcopenia in older adults^14,15^. However, no studies to date have reported the association between ADMA levels and sarcopenia in older adults.

In this study, we examined the relationship between plasma ADMA levels and skeletal muscle mass, grip strength, and sarcopenia in community-dwelling older women. We also analyzed the effects of plasma ADMA levels on the decline in skeletal muscle mass and grip strength over 2 years.

## Results

### Characteristics of the study participants

The characteristics of the entire sample and participants with and without sarcopenia are summarized in Table 1. Overall, the means ± SDs (medians) for age, BMI, SMI, and grip strength were 78.7 ± 6.5 (79.0) years, 22.4 ± 3.2 (22.2) kg/m^2^, 5.63 ± 0.70 (5.63) kg/m^2^, and 21.4 ± 4.4 (21.7) kg, respectively. The number of participants with low SMI (< 5.7 kg/m^2^) and low grip strength (< 18 kg) were 79 (54.9%) and 37 (25.3%), respectively. The number of participants with sarcopenia with both low SMI and low grip strength was 32 (22.2%). None of the participants had low serum albumin levels (< 4.0 g/dl). The mean ± SD (median) plasma ADMA level was 0.48 ± 0.10 (0.47) μM.

**Table 1:** Characteristics of the study participants (n = 144).

	Overall (n = 144)	Participants without sarcopenia (n = 112)	Participants with sarcopenia (n = 32)	*P*
Age, year	78.7 ± 6.5 (79.0)	77.4 ± 6.1 (78.0)	83.3 ± 6.0 (83.0)	< 0.001^a^
Height, cm	149.6 ± 5.9 (149.4)	150.8 ± 5.5 (150.1)	145.1 ± 5.4 (146.2)	< 0.001^a^
Weight, kg	50.1 ± 8.1 (50.1)	51.8 ± 7.7 (50.9)	44.0 ± 6.5 (44.0)	< 0.001^a^
BMI, kg/m^2^	22.4 ± 3.2 (22.2)	22.8 ± 3.2 (22.6)	20.9 ± 3.0 (21.6)	0.004^1a^
Low BMI (< 18.5 kg/m^2^), n (%)	14 (9.7)	6 (5.4)	8 (25.0)	0.003^c^
SMI, kg/m^2^	5.63 ± 0.70 (5.63)	5.84 ± 0.59 (5.78)	4.88 ± 0.52 (4.83)	< 0.001^a^
Low SMI (< 5.7 kg/m^2^), n (%)	79 (54.9)	47 (42.0)	32 (100.0)	< 0.001^c^
Grip strength, kg	21.4 ± 4.4 (21.7)	23.0 ± 3.5 (23.2)	15.8 ± 2.2 (16.4)	< 0.001^a^
Low grip strength (< 18 kg), n (%)	37 (25.3)	5 (4.4)	32 (100.0)	< 0.001^c^
Current and previous smoker, n (%)	11 (7.6)	9 (8.0)	2 (6.3)	1.000^c^
Habitual alcohol drinking (every day and sometimes), n (%)	40 (27.8)	36 (32.1)	4 (12.5)	0.042^c^
Current medication status				
Antihypertension	70 (48.6)	52 (46.4)	18 (56.3)	0.423^c^
Antidyslipidemia	43 (29.9)	35 (30.7)	8 (25.0)	0.662^c^
Serum albumin, mg/ml	4.4 ± 0.26 (4.4)	4.4 ± 0.3 (4.4)	4.3 ± 0.3 (4.3)	0.015^a^
eGFR, ml/min/1.73 m^2^	63.5 ± 13.2 (63.4)	64.1 ± 11.4 (65.1)	61.7 ± 18.4 (61.1)	0.487^a^
Serum TNF-α, pg/ml	1.69 ± 0.68 (1.56)	1.61 ± 0.64 (1.47)	1.97 ± 0.77 (1.79)	0.008^b^

^a^Unpaired *t*-test.

^b^Mann–Whitney U test.

^c^Chi-squired test.

Continuous variables are expressed as average ± SD (median). Categorical variables are expressed as numbers (%).

*BMI*, body mass index; *eGFR*, estimated glomerular filtration rate; *SMI*, skeletal muscle mass; *TNF*, tumor necrosis factor.

**Figure 1:**
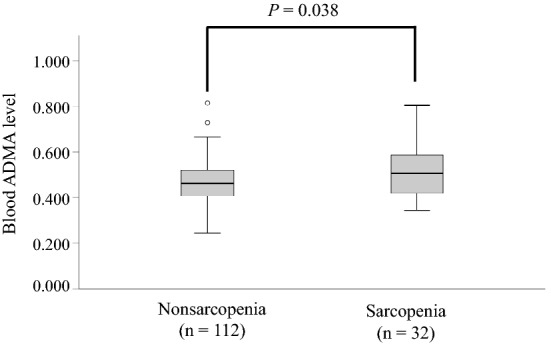
Differences in plasma ADMA levels between older women with and without sarcopenia. Plasma ADMA levels were compared between study participants with and without sarcopenia using the unpaired t-test. Boxplots express medians, interquartile ranges, and ranges. Circles indicate outliers. ADMA, asymmetric dimethylarginine.

Age and the incidence of low BMI (18.5 kg/m^2^) were significantly higher in the sarcopenia group than in the nonsarcopenia group (83.3 ± 6.0 vs. 77.4 ± 6.1 years, *p* < 0.001; 25.0% vs. 5.4%, *p* = 0.003, respectively). Compared with the nonsarcopenia group, serum albumin levels were significantly lower (4.3 ± 0.3 vs. 4.4 ± 0.3, *p* = 0.015) and serum TNF-α levels were significantly higher (1.97 ± 0.77 vs. 1.61 ± 0.64, *p* = 0.008) in the sarcopenia group. Plasma ADMA levels were significantly higher in the sarcopenia group than in the nonsarcopenia group (0.51 ± 0.12 vs. 0.47 ± 0.09, *p* = 0.038; Fig. 1).

### Differences in skeletal muscle mass indices, grip strength, and incidence of sarcopenia according to plasma ADMA levels

Because we found an association between sarcopenia and plasma ADMA level in the comparison between nonsarcopenia and sarcopenia groups, the cutoff plasma ADMA level for sarcopenia was defined using an ROC curve and the Youden Index. This was estimated at 0.57 μM (area under the curve, 0.604; sensitivity, 37.5%; specificity 87.7%). Based on this cutoff value, the participants were categorized into low and high plasma ADMA level groups (Table 2). In the higher ADMA level group, the average grip strength was significantly lower than that in the lower ADMA level group (19.6 ± 3.7 vs. 21.8 ± 4.5, *p* = 0.023). No difference in average SMI was found between the two groups (*p* = 0.199). The incidence of sarcopenia was significantly greater in the higher ADMA level group than in the lower ADMA level group (48.0% vs. 16.8%, *p* = 0.002).

**Table 2:** Differences in skeletal muscle mass indices, grip strength, and incidence of sarcopenia according to plasma ADMA levels in older women.

	Lower ADMA, < 0.57 μM (n = 119)	Higher ADMA, ≥ 0.57 μM (n = 25)	*P*
Plasma ADMA, µM	0.45 ± 0.07	0.64 ± 0.07	< 0.001^a^
SMI, kg/m^2^	5.66 ± 0.68	5.46 ± 0.78	0.199^a^
Low SMI (< 5.7 kg/m^2^), n (%)	64 (53.8)	15 (60.0)	0.661^b^
Grip strength, kg	21.8 ± 4.5	19.6 ± 3.7	0.023^a^
Low grip strength (< 18 kg), n (%)	25 (21.0)	12 (48.0)	0.010^b^
Sarcopenia, n (%)	20 (16.8)	12 (48.0)	0.002^b^

Continuous variables are expressed as average ± SD. Categorical variables are expressed as number (%).

*ADMA* asymmetric dimethylarginine, *SMI* skeletal muscle mass.

^a^Unpaired *t*-test.

^b^Chi-squired test.

### Logistic regression analysis of associations between sarcopenia and plasma ADMA levels

Table 3 shows the results of logistic regression analysis of the relationship between sarcopenia and plasma ADMA levels. The crude OR of a higher plasma ADMA level (≥ 0.57 μM) was 4.57 (95% CI 1.82–11.47; *p* = 0.001). The age- and BMI-adjusted OR for higher plasma ADMA level was 4.01 (95% CI 1.35–11.93: *p* = 0.012). This association between sarcopenia and plasma ADMA levels remained significant after adjustment for all relevant variates, including age; BMI; alcohol consumption; use of antihypertension and antidyslipidemia medications; and serum albumin, eGFR, and TNF-α levels (OR, 4.45; 95% CI 1.13–14.96; *p* = 0.016). Thus, higher plasma ADMA levels were associated with the presence of sarcopenia in community-dwelling older Japanese women.

**Table 3:** Logistic regression analysis of the relationship between sarcopenia and plasma ADMA levels in older women.

	Odds ratio	95% CI (lower, upper)	*P*
Crude			
Higher plasma ADMA levels (≥ 0.57 µM)	4.57	1.82, 11.47	0.001
Age, BMI-adjusted model			
Higher plasma ADMA levels (≥ 0.57 µM)	4.01	1.35, 11.93	0.012
Age (increment of 5 years)	2.44	1.53, 3.90	< 0.001
Low BMI (< 18.5 kg/m^2^)	2.33	0.59, 9.17	0.226
Multivariate model			
Higher plasma ADMA levels (≥ 0.57 µM)	4.45	1.13, 14.96	0.016
Age (increment of 5 years)	2.30	1.39, 3.80	0.001
Low BMI (< 18.5 kg/m^2^)	2.75	0.61, 12.33	0.187
Current and previous smoker	1.64	0.22, 12.30	0.632
Habitual alcohol drinking (every day and sometimes)	0.39	0.10, 1.51	0.173
Current use of antihypertension medications	1.27	0.45, 3.63	0.652
Current use of antidyslipidemia medications	0.71	0.24, 2.09	0.530
Lower serum albumin levels (< 4.4 g/dl)	2.24	0.84, 5.96	0.106
eGFR (< 45, 45–60, ≥ 60 ml/min/1.73 m^2^)	1.20	0.86, 1.66	0.277
Higher TNF-α levels (≥ 1.56 pg/ml)	1.97	0.69, 5.63	0.206

*ADMA* asymmetric dimethylarginine, *BMI* body mass index, *eGFR* estimated glomerular filtration rate, *SMI* skeletal muscle mass, *TNF* tumor necrosis factor.

### A higher plasma ADMA level was associated with skeletal muscle loss after 2 years

Associations between plasma ADMA levels and reductions in the SMI and grip strength of the 85 participants who underwent our follow-up examination in 2017 were analyzed. Differences in the measured parameters among the follow-up study participants are presented in Supplementary Table 1.

**Figure 2:**
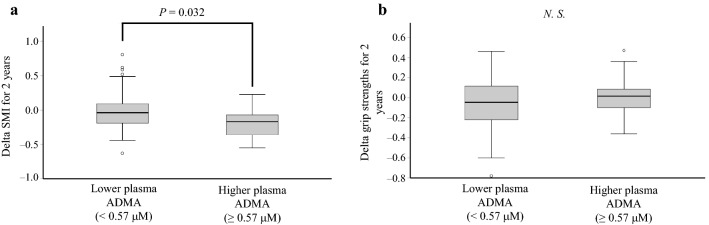
Comparison of skeletal muscle loss over 2 years in older women according to plasma ADMA levels. Reductions in (**a**) SMI and (**b**) grip strength over 2 years were compared according to plasma ADMA levels using the Mann–Whitney U test. Boxplots express medians, interquartile ranges, and ranges. Circles indicate outliers. ADMA, asymmetric dimethylarginine; SMI, skeletal muscle mass index.

No significant differences were found in BMI, SMI, or grip strength between 2015 and 2017. However, a significantly greater reduction in SMI over the 2 years was observed in the higher plasma ADMA level group than in the lower plasma ADMA level group, with medians (interquartile ranges) of − 0.17 (0.32) and − 0.04 (0.28), respectively (*p* = 0.032; Fig. 2a). However, there was no difference in the reduction in grip strength over 2 years between the two groups (*p* = 0.406; Fig. 2b).

## Discussion

In this study, we found that increased plasma ADMA levels were significantly associated with low grip strength and sarcopenia in community-dwelling older women. In addition, increased plasma ADMA levels were related to skeletal muscle loss over 2 years. This is the first study to reveal a relationship between ADMA levels and sarcopenia in community-dwelling older women.

Although the mechanism underlying the association between ADMA levels and sarcopenia remains unclear, decreased NO availability is known to cause a loss of muscle mass and muscle dysfunction. Age-related decreases in NO synthase levels in skeletal muscles increase myofibril degradation and muscle wasting through protease activity^16^. Research in mice has found that NO stimulates satellite cell proliferation, increases satellite cell number in myofibers, and recovers skeletal muscle function^17^. Supplementation with sodium nitrite has also been shown to improve motor function, including grip strength, in aged mice^18^. The same study also found that the levels of inflammatory cytokines, such as interleukin-1β and TNF-α, in muscles were reduced by the nitrite supplementation, indicating that NO improves motor function through the reduction of inflammatory cytokines. In this study, serum TNF-α levels in older women with sarcopenia showed a significantly greater increase than in those without sarcopenia, suggesting that chronic inflammation is also associated with sarcopenia. However, when serum TNF-α levels were excluded from the multivariate model, our logistic regression analysis revealed that plasma ADMA levels had a stronger association with sarcopenia than TNF-α. Recently, ADMA was identified as a causal factor in muscle wasting in a mouse model of cachexia^19^. The study revealed that ADMA impairs muscle protein synthesis through the inhibition of mitochondrial function. Thus, higher ADMA levels may contribute to the development of sarcopenia through these processes.

Physical exercise has been reported to reduce plasma ADMA levels^20–22^. This could create a feedback loop in which a sedentary lifestyle leads to sarcopenia and increased plasma ADMA levels, and the increased plasma ADMA levels and sarcopenia accelerate the ongoing reductions in grip strength and skeletal muscle mass.

Although a significant reduction in SMI was observed in participants with higher plasma ADMA levels over the 2 years, a reduction in grip strength was not observed in our analysis. One possible cause is that the individuals measuring grip strength were different at baseline and 2 years later. Additionally, the sample size of the 2-year follow-up analysis was small (n = 85); therefore, the interindividual error may have affected the results. Werdyani et al*.*^23^ reported that an increased ADMA level is associated with the reduction in hand grip strength over 10 years. Subsequently, a longer observation period may be needed.

We determined the cutoff plasma ADMA level for sarcopenia to be 0.57 μM. The means ± SDs of plasma ADMA levels in the low and high ADMA level groups were 0.45 ± 0.07 and 0.64 ± 0.07, respectively. A previous study showed significant differences in grip strength and gait speed in a comparison between low (mean ± SD of serum ADMA was 0.39 ± 0.002 μM) and high (0.52 ± 0.005 μM) serum ADMA level groups of older adults, although no differences were observed between low ADMA and intermediate ADMA level (0.45 ± 0.01 μM) groups^14^. While comparing absolute values of ADMA levels between studies is difficult because levels vary depending on the measurement method and the reference range is broad, the difference in mean ADMA levels between the low and high groups in our study was similar to that found in a previous study^24^.

This study had several limitations. First, the cross-sectional design and small sample size for the 2-year follow-up did not allow us to infer the causal relationship between sarcopenia and ADMA. Second, the study was limited by the sample size (cross-sectional study, n = 144; 2-year follow-up study, n = 85) and the availability of only female participants because the sample comprised women who chose to undergo our annual health examination. Therefore, further research should use prospective designs and larger sample sizes to clarify the relationship between sarcopenia and ADMA. Third, blood samples were stored at − 30 °C, which may have affected ADMA levels. Fourth, we used bioimpedance analysis to measure skeletal muscle mass. Although the gold standard for body composition measurement is dual-energy X-ray absorptiometry, the criteria for sarcopenia include SMI measured by bioimpedance analysis^1^. Fifth, the incidence of sarcopenia may have been underestimated in this study as participants’ gait speeds could not be measured owing to space limitations. However, the prevalence of sarcopenia in community-dwelling older Japanese women has been reported to be 16.5% (n = 1851) in a previous study^25^. Because the prevalence of sarcopenia in the current study was 22.2%, we seem to not have underestimated the prevalence of sarcopenia, but rather may have obtained a slightly higher prevalence of sarcopenia than that in the general population. Finally, previous studies have found an association between vitamin D and ADMA levels and sarcopenia; however, we could not use vitamin D as a covariate in this study owing to the lack of data^26,27^. Hence, additional analysis with vitamin D is required.

In conclusion, this study found that higher plasma ADMA levels in community-dwelling older Japanese women are associated with sarcopenia and a decline in skeletal muscle mass over 2 years. Reducing plasma ADMA levels is therefore important for the prevention of sarcopenia and frailty. Further research is needed to establish strategies for reducing plasma ADMA levels.

## Methods

### Study participants

The study participants were community-dwelling older women who attended voluntary lunch events held in seven community centers in Nishinomiya City, Hyogo Japan. The inclusion criteria for this study were receipt of an annual health examination in community centers before lunch events between September 2015 and October 2015. Individuals who were < 65 years old, those with diabetes, cardiovascular diseases, or rheumatism, and those with an estimated glomerular filtration rate (eGFR) of < 30 ml/min/1.73 m^2^ were excluded. Owing to the number of men being too small (n = 16), these participants were excluded from the analysis. A further seven patients were excluded because of missing anthropometric or blood data. Of 232 older adults who participated in health examinations, 144 were included in our analysis. Of these, 85 underwent our health examination in 2017 and were included in the analysis examining the association between plasma ADMA levels and reductions in muscle mass and muscle strength over 2-years. The grip strength of one participant was not measured in the health examination conducted in 2017.

### Ethics statement

This study was conducted in accordance with the 2013 revision of the Declaration of Helsinki. The study protocol was approved by the Ethics Committee of Mukogawa Women’s University (approval number 15-04). All participants provided written informed consent for participation in this study and publication of the article. The sample size was determined based on the number of participants who underwent the health examination.

### Anthropometric and laboratory measurements

Blood samples were collected in the morning (from 10:00 to 11:00 AM). The blood samples of 118 participants were collected following overnight fasting, whereas 26 participants self-reported eating something beforehand. Serum albumin, creatinine, and tumor necrosis factor (TNF)-α levels were measured by a clinical laboratory (LSI Medience Corp., Tokyo, Japan) using the improved bromcresol method, enzymatic method, and chemiluminescent enzyme immunoassay, respectively. eGFR levels were calculated based on age and serum creatinine levels.

Anthropometric measurements were then performed and included height, weight, skeletal muscle mass, and grip strength. Weight and limb skeletal muscle mass were measured by bioimpedance analysis using the InBody 430 body composition analyzer (BioSpace Inc., Cerritos, CA, USA). Body mass index (BMI) was calculated as weight (kg) divided by height (m) squared. Skeletal muscle mass index (SMI) was calculated as limb skeletal muscle mass (kg) divided by height (m) squared. The grip strength of the dominant hand was measured with the patient being in a standing position using a grip strength dynamometer (GRIP-D, Takei Scientific Instrument Co. Ltd., Japan). The higher value (kg) measure from two trials was used for the analysis. These anthropometric data were collected by well-trained staff .

### Measurement of plasma ADMA levels

Plasma samples were obtained by centrifugation of the participants’ blood samples using heparin and stored at − 30 °C. Plasma ADMA levels were measured in our laboratory using a competitive enzyme-linked immunosorbent assay, according to previously described methods^28^. Briefly, blood samples were pretreated with N-succinimidyl 3-maleimidobenzoate (SMB) and mixed with monoclonal anti-SMB-ADMA antibody. The mixtures were added to plates coated with ADMA-SMB-bovine serum albumin conjugates and incubated overnight at 4 °C. After labeling with horseradish peroxidase-bound secondary antibodies, ADMA levels were detected by chemiluminescence using o-phenylenediamine. The cross-reactivity of this ELISA system with L-arginine was < 0.01%.

### Assessment of sarcopenia

Sarcopenia is indicated by low muscle strength, low physical performance, and low height-adjusted muscle mass. We measured grip strength and skeletal muscle mass by bioimpedance analysis during the health examinations. In this study, sarcopenia was defined as a grip strength of < 18 kg and SMI of < 5.7 kg/m^2^ based on the definition of the Asian Working Group for Sarcopenia 2019 Consensus^3^.

### Other demographic variables

Other demographic data including age, current medication status (antihypertension and antidislipiemia), smoking status (current smoker, previous smoker, or nonsmoker), and drinking habits (every day, sometimes, or never) were obtained.

### Statistical analysis

Quantitative variables are expressed as the mean ± standard deviation (SD) (median). Categorical variables are expressed as numbers (percentages). We categorized the participants into those with and thosewithout sarcopenia. The two groups were compared using unpaired *t*-tests for quantitative variables with normal distributions, Mann–Whitney U tests for quantitative variables with non-normal distributions, and chi-square tests for categorical variables. Because there is no established cutoff value for plasma ADMA levels indicative of sarcopenia, the cutoff was estimated using a receiver-operating characteristic (ROC) curve and the Youden Index. The odds ratio (OR) and 95% confidence interval (CI) for sarcopenia were determined by logistic regression analysis. The covariates of multivariate-adjusted logistic regression analysis were age (categorized by an increment of 5 years), lower BMI (< 18.5 kg/m^2^), smoking (current and previous), habitual alcohol consumption (every day and sometimes), current medication for hypertension or dyslipidemia, and high serum TNF-α levels (≥ 1.56 pg/ml of the median value). Low serum albumin level was also a covariate; however, none of the participants had low serum albumin levels (< 4.0 g/dl)^29^. Therefore, the median was used as the reference value (< 4.4 g/dl). To examine the associations between plasma ADMA levels and reduced muscle mass and muscle strength, the within-subject differences in SMI and grip strength between 2015 and 2017 were compared between those with lower plasma ADMA levels and those with higher plasma ADMA levels using Mann–Whitney U tests. All statistical data were analyzed using SPSS v. 26.0 (IBM Corp., Armonk, NY, USA) software. Two-tailed *p* values of < 0.05 were considered statistically significant.

### Consent to participate

Informed consent was obtained from all individual participants included in the study. Informed consent was obtained from legal guardians. Written informed consent was obtained from all participants.

## Supplementary Information

Supplementary Table 1.

## Data Availability

Data generated or analyzed in this study is available from the corresponding author on reasonable request.

## References

[1] Chen LK (2020). Asian Working Group for Sarcopenia: 2019 consensus update on sarcopenia diagnosis and treatment. J. Am. Med. Dir. Assoc..

[2] Beaudart C (2019). Malnutrition as a strong predictor of the onset of sarcopenia. Nutrients.

[3] Chen H, Ma J, Liu A, Cui Y, Ma X (2020). The association between sarcopenia and fracture in middle-aged and elderly people: A systematic review and meta-analysis of cohort studies. Injury.

[4] Cruz-Jentoft AJ (2019). Sarcopenia: Revised European consensus on definition and diagnosis. Age Ageing.

[5] Aggio DA (2016). Cross-sectional associations of objectively measured physical activity and sedentary time with sarcopenia and sarcopenic obesity in older men. Prev. Med..

[6] Bai T (2020). Sarcopenia is associated with hypertension in older adults: A systematic review and meta-analysis. BMC Geriatr..

[7] Han CH, Chung JH (2018). Association between sarcopenia and tooth loss. Ann. Geriatr. Med. Res..

[8] Pacifico J (2020). Prevalence of sarcopenia as a comorbid disease: A systematic review and meta-analysis. Exp. Gerontol..

[9] Mankhong S (2020). Experimental models of sarcopenia: bridging molecular mechanism and therapeutic strategy. Cells.

[10] Abbatecola AM (2012). Pulse wave velocity is associated with muscle mass decline: Health ABC study. Age.

[11] Yamanashi H (2018). Association between atherosclerosis and handgrip strength in non-hypertensive populations in India and Japan. Geriatr. Gerontol. Int..

[12] Vallance P, Leone A, Calver A, Collier J, Moncada S (1992). Accumulation of an endogenous inhibitor of nitric oxide synthesis in chronic renal failure. Lancet.

[13] Willeit P (2015). Asymmetric dimethylarginine and cardiovascular risk: Systematic review and meta-analysis of 22 prospective studies. J. Am. Heart Assoc..

[14] Obayashi K (2016). Association of serum asymmetric dimethylarginine with muscle strength and gait speed: A cross-sectional study of the HEIJO-KYO cohort. J. Bone Miner. Res..

[15] Pajek M, Jerman A, Osredkar J, Ponikvar JB, Pajek J (2018). Association of uremic toxins and inflammatory markers with physical performance in dialysis patients. Toxins.

[16] Samengo G (2012). Age-related loss of nitric oxide synthase in skeletal muscle causes reductions in calpain S-nitrosylation that increase myofibril degradation and sarcopenia. Aging Cell.

[17] Buono R (2012). Nitric oxide sustains long-term skeletal muscle regeneration by regulating fate of satellite cells via signaling pathways requiring Vangl2 and cyclic GMP. Stem Cells.

[18] Justice JN (2015). Sodium nitrite supplementation improves motor function and skeletal muscle inflammatory profile in old male mice. J. Appl. Physiol..

[19] Kunz HE (2020). Methylarginine metabolites are associated with attenuated muscle protein synthesis in cancer-associated muscle wasting. J. Biol Chem..

[20] Riccioni G (2015). Physical exercise reduces synthesis of ADMA, SDMA, and L-Arg. Front. Biosci. (Elite Ed).

[21] Shimomura M (2021). Relationship between plasma asymmetric dimethylarginine and nitric oxide levels affects aerobic exercise training-induced reduction of arterial stiffness in middle-aged and older adults. Phys. Act. Nutr..

[22] Tanahashi K (2014). Plasma ADMA concentrations associate with aerobic fitness in postmenopausal women. Life Sci..

[23] Werdyani S (2022). Metabolomic signatures for the longitudinal reduction of muscle strength over 10 years. Skelet. Muscle.

[24] Németh B (2017). The issue of plasma asymmetric dimethylarginine reference range – A systematic review and meta-analysis. PLoS ONE.

[25] Kitamura A (2021). Sarcopenia: Prevalence, associated factors, and the risk of mortality and disability in Japanese older adults. J. Cachexia Sarcopenia Muscle.

[26] Choi HR (2017). Association between vitamin D status and asymmetric dimethylarginine (ADMA) concentration in the Korean elderly population. Maturitas.

[27] Bollen SE (2022). The vitamin D/Vitamin D receptor (VDR) axis in muscle atrophy and sarcopenia. Cell Signal..

[28] Yokoro M (2012). Development of an enzyme-linked immunosorbent assay system for the determination of asymmetric dimethylarginine using a specific monoclonal antibody. Biosci. Biotechnol. Biochem..

[29] Uemura K, Doi T, Lee S, Shimada H (2019). Sarcopenia and low serum albumin level synergistically increase the risk of incident disability in older adults. J. Am. Med. Dir. Assoc..

