# Supplementary material for: Association between asymmetric dimethylarginine and sarcopenia in community-dwelling older women
Source: Sci Rep. 2023 Apr 4;13:5510. doi: 10.1038/s41598-023-32046-0 (PMC10073180; doi:10.1038/s41598-023-32046-0)
Supplement: Supplementary file 1 — Supplementary Table 1. [file 41598_2023_32046_MOESM1_ESM.pdf]

Association between asymmetric dimethylarginine and sarcopenia in community-dwelling older women

Miyuki Yokoro[1,2], Naoto Otaki[2,3], Megumu Yano[2,4], Tomomi Imamura[2,5], Norikazu Tanino[2], Keisuke Fukuo[2,3]

1 Department of Dietary Life and Food Sciences, Junior College Division, Mukogawa Women's University, 2 Research Institute for Nutrition Sciences, Mukogawa Women's University, 3 Department of Food Sciences and Nutrition, School of Food Sciences and Nutrition, Mukogawa Women's University, 5 Department of Innovative Food Sciences, School of Food Sciences and Nutrition, Mukogawa Women's University, Japan

Correspondence: Keisuke Fukuo

Department of Food Sciences and Nutrition, School of Food Sciences and Nutrition, Mukogawa Women's University, 6-46 Ikebiraki-cho, Nishinomiya, Hyogo 663-8558, Japan.

Email: fukuo@mukogawa-u.ac.jp

TEL and FAX: +81-798-45-9922

Supplementary Table 1. Differences in study participant characteristics between 2015 and 2017

	n	Baseline in 2015	2 years follow-up in 2017	***P***[1)]
Age, year	85	78.5 ± 6.1 (79.0)	80.5 ± 6.1 (81.0)	<0.001
Height, cm	85	149.0 ± 6.1 (149.1)	148.5 ± 6.3 (148.8)	<0.001
Weight, kg	85	49.7 ± 7.8 (49.8)	49.3 ± 8.1 (49.8)	0.627
BMI, kg/m^2	85	22.4 ± 3.0 (22.3)	22.6 ± 3.1 (22.3)	0.027
SMI, kg/m^2	85	5.70 ± 0.66 (5.70)	5.64 ± 0.69 (5.66)	0.067

1) Paired t-test.

BMI, body mass index; SMI, skeletal muscle index.
